# A Case of Rhabdomyolysis in a Young, Morbidly Obese, Asthmatic Woman With COVID-19

**DOI:** 10.7759/cureus.28950

**Published:** 2022-09-08

**Authors:** Johnafaye Mariano, George A MacLaren

**Affiliations:** 1 Internal Medicine, Riverside Regional Medical Center, Newport News, USA

**Keywords:** covid-19 and rhabdomyolysis, covid-19 and asthma, covid-19 and obesity, elevated creatine kinase, transaminitis, rhabdomyolysis, asthma, covid-19

## Abstract

COVID-19 is a respiratory disease that has been shown to have extrapulmonary manifestations. One association with COVID-19 is rhabdomyolysis, which is defined as the breakdown of skeletal muscles. There have been increasing reports of rhabdomyolysis in obese, middle-aged male COVID-19 patients, but limited published cases affecting young adult females. This case discusses the early presentation of rhabdomyolysis in a young, morbidly obese, asthmatic woman with COVID-19.

A 28-year-old, unvaccinated, African American female with past medical history of asthma, tobacco abuse, and a BMI of 46 initially presented to the emergency department with a complaint of fever, cough, and shortness of breath for two days. She was initially diagnosed with an asthma exacerbation and was treated symptomatically, but her symptoms persisted despite treatment. She began to experience myalgias the next day, followed by bilateral lower extremity weakness and dark urine two days later. Urinalysis revealed gross hematuria, 2-4 red blood cells per high-power field, 100 mg/dL protein, >8.0 mg/dL urobilinogen, and 0-2 hyaline casts. Alanine aminotransferase (ALT), aspartate aminotransferase (AST), and creatine kinase (CK) levels were noted to be elevated. Her subsequent COVID-19 test was positive, and both blood and respiratory cultures were negative. She was diagnosed with rhabdomyolysis which was likely secondary to COVID-19. Her CK, ALT, and AST levels normalized after two weeks with the resolution of rhabdomyolysis, but she continued to have persistent COVID-19 infection and deteriorating respiratory status. She eventually required mechanical ventilation on day 20 and passed away on day 59 of hospitalization.

Rhabdomyolysis is an infrequent finding that can be associated with COVID-19. It has been increasingly reported in middle-aged obese male patients but is far less common in younger females. The presence of elevated CK has been associated with higher mortality among COVID-19 patients, but current literature demonstrates that the majority of these patients are older males. It is imperative to recognize and treat rhabdomyolysis in all patients, particularly younger females, to help mitigate the comorbidities of COVID-19.

## Introduction

COVID-19 is a respiratory disease caused by the novel SARS-CoV-2 virus that has resulted in a global pandemic and millions of deaths in the past two and a half years. Initially detected in Wuhan, China in December 2019, the primary reservoir of SARS-CoV-2 are bats and it is transmitted human to human via respiratory droplets [1]. The pathophysiology of COVID-19 involves the spike glycoprotein (S) of SARS-CoV-2 attaching to the angiotensin-converting enzyme 2 (ACE2) receptor of host type 2 pneumocytes, followed by cleavage and activation of the ACE2 receptor via type 2 transmembrane serine protease 2 (TMPRSS2), allowing fusion of SARS-CoV-2 with respiratory epithelial cells [2]. The resulting release of proinflammatory cytokines, such as IL-6, IL-10, and TNF-α, can lead to pneumonia, acute respiratory distress syndrome (ARDS), and multi-organ dysfunction syndrome (MODS) [2]. Although the main target of SARS-CoV-2 is the respiratory system, it has been associated with a myriad of extrapulmonary symptoms including cardiovascular, hematologic, neurologic, and renal systems [3]. Notably, infrequent cases of rhabdomyolysis associated with COVID-19 have been emerging since the onset of the global pandemic [4-9]. A systematic review of case reports over the past two years showed an estimated incidence of rhabdomyolysis between 0.2 and 2.2% among hospitalized COVID-19 patients, in which the median age was 50 years old and 77% comprised males [9]. Among these patients, 36% required mechanical ventilation and 30% died. A 2021 meta-analysis of observational studies reviewing 2471 patients reported an incidence of elevated creatine kinase (CK) of 17%, and it was found that an elevated CK level is associated with a 49% probability of having severe COVID-19 infection or death compared to 24% probability in patients who had a normal CK level [10]. Rhabdomyolysis, which is defined as skeletal muscle breakdown resulting in the release of intracellular components and myoglobin, can have a variety of causes. The most common etiologies of rhabdomyolysis include trauma secondary to crush injury, immobilization, overexertion, and exogenous toxins such as ethanol, illicit drugs, and prescribed medications (particularly statin drugs) [11]. Viruses have also been reported as causative agents, including influenza A & B, enteroviruses, and the original SARS-CoV that was responsible for the SARS outbreak in 2003 [7,11-13]. Potential mechanisms for viral rhabdomyolysis include direct viral invasion of muscles, cytokine storm within muscle tissue, and destruction of the muscle cell membrane due to circulating toxins [14]. However, the exact mechanism of rhabdomyolysis induced by SARS-CoV-2 is still unknown [14]. COVID-associated rhabdomyolysis may have an initial or late presentation in the disease course [7,8,13,15]. Diagnosis of rhabdomyolysis is made if the serum CK, an enzyme used as an indicator for muscle damage, is elevated above 1000 U/L [6]. Severe rhabdomyolysis is noted if serum CK is above 5000 U/L [6]. Serum myoglobin is not routinely tested in the setting of rhabdomyolysis due to its short half-life of two to three hours, and it is rapidly excreted and metabolized to bilirubin [16,17]. A small amount of bilirubin is excreted as urobilinogen, which can be detected by urinalysis [16,17]. Myoglobin can be detected in the urine if the serum concentration exceeds 1.5 mg/dL, and visible changes in the urine can be seen when urine myoglobin levels reach 100 to 300 mg/dL [16,17]. In this case report, we will explore the clinical course of a young, morbidly obese, asthmatic woman, who contracted COVID-19 and was noted to have rhabdomyolysis as an initial presentation.

## Case presentation

**Figure 1:**
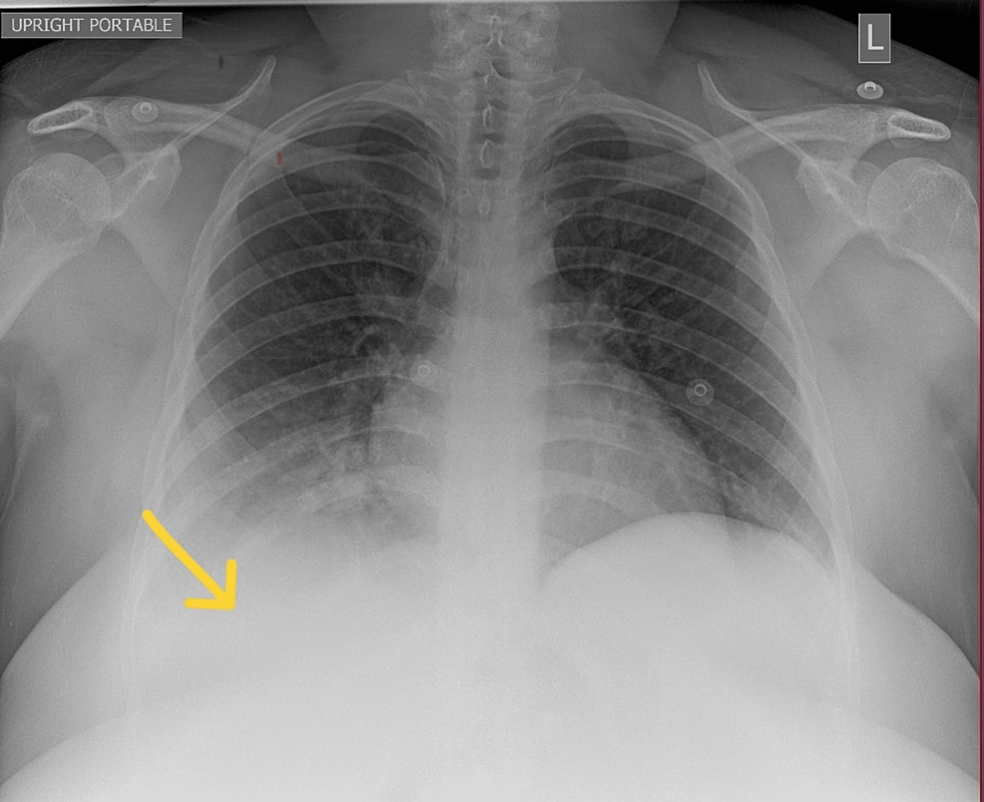
Chest X-ray reveals consolidation in the right lung base with small right pleural effusion, which is concerning for lobar pneumonia. The arrow is pointing toward the area of consolidation.

**Figure 2:**
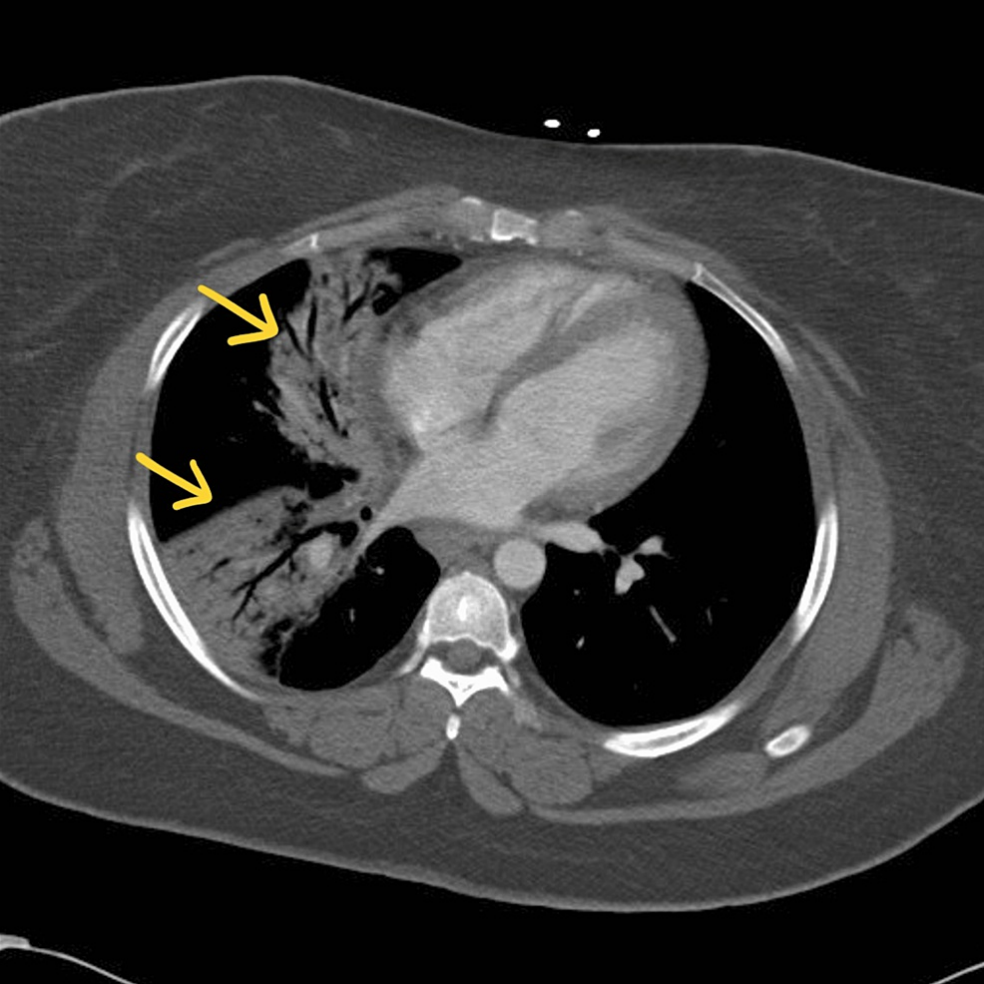
Chest CT taken on day 1 of hospitalization. Imaging reveals right lower and right middle lobe airspace disease consistent with pneumonia. No evidence of pulmonary embolism. The arrows point toward the areas of consolidation.

**Figure 3:**
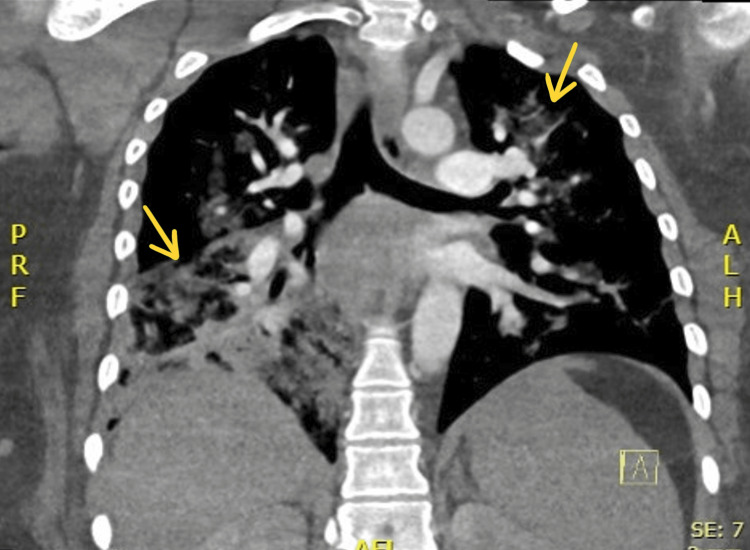
CT pulmonary angiogram taken on day 2 of hospitalization. Imaging reveals bilateral pulmonary infiltrates and consolidations, greater in the right lower lobe. The arrows point toward areas of consolidation.

**Figure 4:**
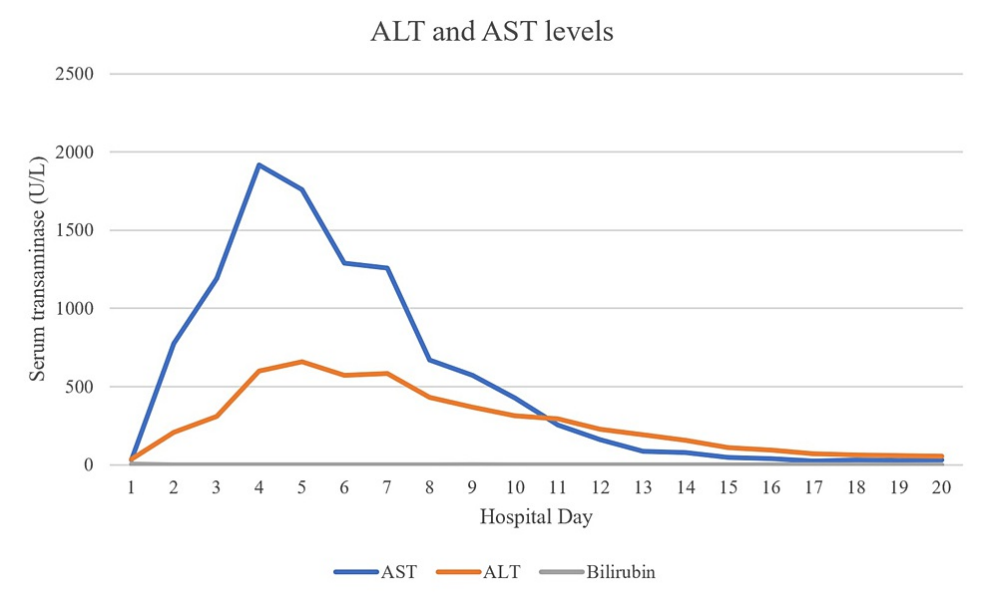
Patient’s aminotransferase levels during the first 20 days of hospitalization. Values beyond 20 days were excluded from this graph since they remained within normal range for the rest of the hospitalization. The reference ranges are 9-32 U/L for AST and 19-25 U/L for ALT in healthy women. AST: aspartate aminotransferase, ALT: alanine aminotransferase

**Figure 5:**
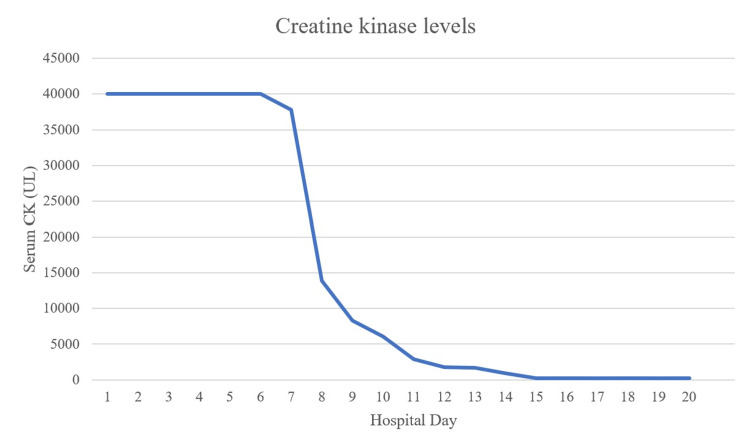
Patient’s creatine kinase levels during the first 20 days of hospitalization. Creatine kinase levels stayed within normal range for the remainder of the hospitalization. The reference ranges are 10-70 U/L for healthy females and 25-90 U/L for healthy males.

**Figure 6:**
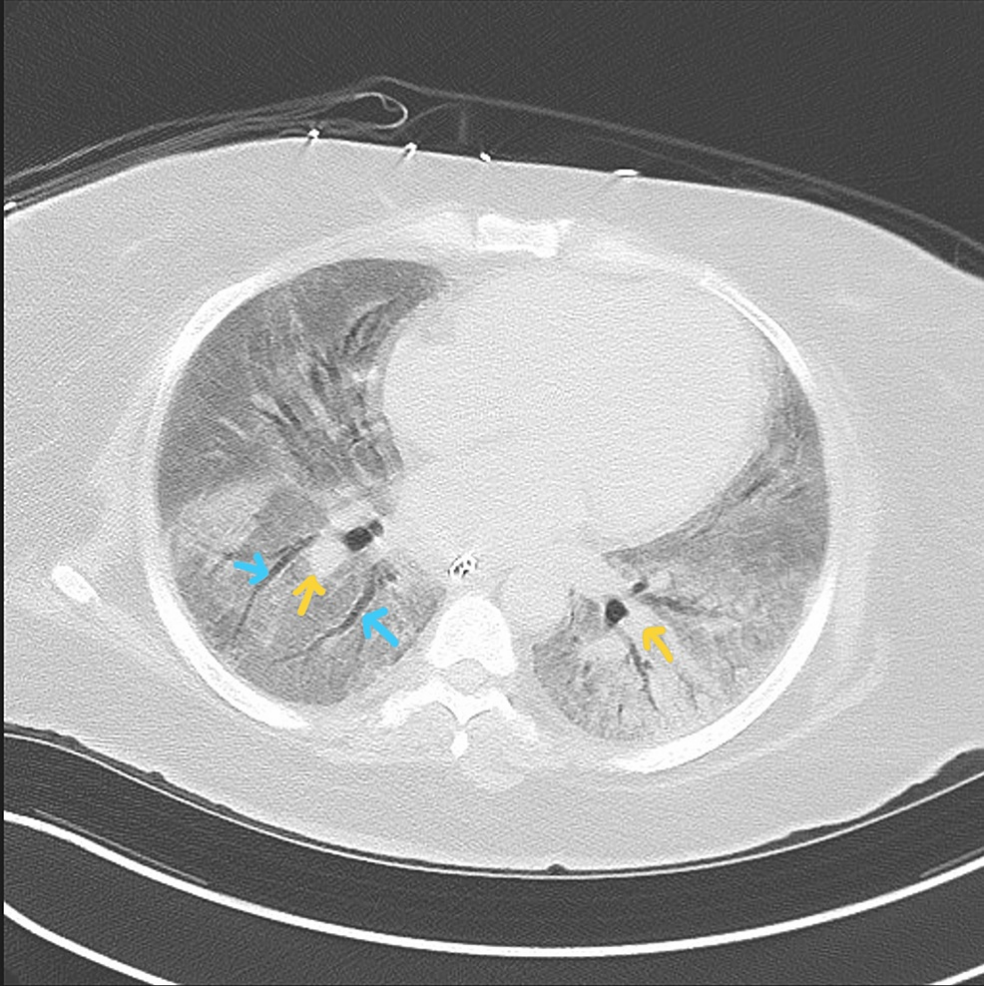
Chest CT taken on day 57 of hospitalization. Imaging reveals diffuse pulmonary fibrosis with ground-glass attenuation, bilateral centrilobular consolidations (yellow arrows) most prominent within the dependent lungs, and traction bronchiectasis (blue arrows).

A 28-year-old, unvaccinated, African American female with a past medical history of asthma, tobacco abuse, and a BMI of 46 initially presented to the emergency department in September 2021 with a complaint of fever, cough, and shortness of breath for two days. Her initial COVID-19 test was negative, and her symptoms were attributed to asthma which was treated with albuterol. Of note, her last documented asthma exacerbation was one month prior to her current presentation. She continued to have a fever with a peak of 104°F along with myalgias and arthralgias which prompted her to return to the ED the following day. Her SpO_2_ was 98%, and chest X-ray showed signs of lobar pneumonia (Figure 1). She was empirically treated as an outpatient with amoxicillin-clavulanic acid. Her symptoms worsened despite treatment, so she returned to the ED two days later with an additional complaint of dark urine and bilateral lower extremity muscle weakness. She was found to have SpO_2_ of 93% with desaturation to 80% when walking, which required treatment with supplemental oxygen at 2 L/min via nasal cannula. Urinalysis revealed gross hematuria, 2-4 red blood cells per high-power field, 100 mg/dL protein, >8.0 mg/dL urobilinogen, and 0-2 hyaline casts; urine hemoglobin and urine myoglobin were not part of the urinalysis test. She was retested for COVID-19 which came back positive, but other viral testing was not performed to rule out alternate viral etiologies. She did meet sepsis criteria at that time for possible superimposed bacterial pneumonia (fever, elevated leukocyte count, tachycardia, elevated respiratory rate, and elevated lactic acid > 2.0), thus necessitating hospital admission. She was empirically treated with a course of levofloxacin and ceftriaxone for pneumonia while respiratory and blood culture results were pending; however, both culture results came back negative a few days later. Antibiotics were discontinued after receiving the negative culture results, and she was started on corticosteroids to address acute respiratory distress. Chest CT on day 1 of hospitalization revealed consolidation in the right middle and right lower lobes, but it was negative for pulmonary embolism (Figure 2). CT pulmonary angiogram on day 2 revealed worsening consolidation and pulmonary infiltrates that had become bilateral, and ground-glass type opacities predominant in the central lungs concerning for COVID-19 pneumonia (Figure 3). Meanwhile, her ALT and AST levels increased exponentially over the duration of 12 hours since admission, with a peak on day 5 (Figure 4). Her CK level was also elevated at > 40,000 U/L during the first six days of hospitalization (Figure 5). The following liver enzymes were within normal limits: ALP (75 U/L) and gamma-glutamyltransferase (GGT) (45 U/L). Total bilirubin (1.6 mg/dL) and international normalized ratio (INR) (0.95) were also within the normal range. Urine culture was negative and repeat testing four days later remained negative. A comprehensive metabolic panel showed creatinine levels between 1.08 and 1.32 mg/dL and glomerular filtration rate (GFR) between 48 and 59 mL/min/1.73 m^2^ corresponding with the timeframe of the transaminitis. Blood ethanol and acetaminophen levels were within normal limits. Urine Legionella antigen, urine drug screen, and hepatitis panel were negative. Her liver ultrasound revealed a normal biliary tract without evidence of fibrosis. Gastroenterology was consulted, and transaminitis was attributed secondary to rhabdomyolysis likely associated with COVID-19. It was suggested that transaminitis would improve once CK level normalized, so conservative management of rhabdomyolysis with intravenous fluids was advised. She was initially unable to receive remdesivir treatment for COVID-19 due to transaminitis, and an IL-6 inhibitor was contraindicated given her underlying sepsis. She did receive corticosteroids and supplemental oxygen for the treatment of COVID-19. After day 8, her CK, ALT, and AST levels started to decrease, but her respiratory status continued to deteriorate despite previous treatment with corticosteroids. Her supplemental oxygen requirement steadily increased from 2 L/min on admission to 30 L/min via high-flow nasal cannula the following week and up to 60 L/min two weeks after admission. She was retested for COVID-19 on day 16 which came back positive. Her respiratory and blood cultures were repeated at the same time, and they remained negative. Given her negative respiratory and blood cultures, her worsening respiratory status was attributed to COVID-19. She was started on remdesivir treatment after resolution of rhabdomyolysis and transaminitis; this was the only available option during her hospitalization in September 2021 given the supply shortages at that time. Maximal medical therapy was reached without improvement of her respiratory status, and she eventually required intubation and mechanical ventilation on day 20. She received propofol, cisatracurium, and epoprostenol while being mechanically ventilated, and she required upwards of 15 cm H2O of positive end-expiratory pressure (PEEP). Attempted ventilator weaning trials were unsuccessful as she rapidly desaturated each time. She developed tongue ulceration after prolonged intubation on day 44, followed by sepsis. CT angiogram of the chest on day 57 revealed diffuse pulmonary fibrosis with associated ground-glass attenuation, bilateral centrilobular consolidations, and traction bronchiectasis (Figure 6). These findings were attributed to the sequelae of severe COVID-19 pneumonia. Her family decided to switch to comfort care, and the patient passed away on day 59.

## Discussion

This case describes how rhabdomyolysis associated with COVID-19 can be an initial transient presentation in a young adult female, in contrast to several reported cases involving middle-aged male patients [6,7,9,14,15]. Although there is one reported case of a 19-year-old female with an initial presentation of rhabdomyolysis, she had a daily strength training routine which likely contributed to muscle damage, whereas our patient did not have any preceding history of crush injury or strenuous exercise prior to rhabdomyolysis [5]. Our patient did eventually require sedation and immobilization on day 20 for mechanical ventilation; however, this would not explain her elevated CK levels on admission. If immobilization was the cause of rhabdomyolysis, then CK level would have risen after she was placed on mechanical ventilation. Furthermore, her negative urine drug screen, normal blood ethanol level, and negative medication history exclude exogenous toxins as the etiology of rhabdomyolysis. Her initial elevated CK of >40,000 U/L on admission is notable since this could have been a prognostic indicator for her poor outcome. According to two systematic reviews of case reports, an elevated CK level with or without overt rhabdomyolysis was associated with higher severity of COVID-19 infection and death [9,10]. Although there is no causal relationship between elevated CK level and poor prognosis, perhaps it could have been used as a sign to initiate more aggressive treatments early on to help mitigate further complications.

Her urinalysis findings are also pertinent in the setting of rhabdomyolysis. Although urine hemoglobin and urine myoglobin were not measured, the presence of elevated urinary protein and urobilinogen may indicate urinary excretion of myoglobin metabolites. As a brief review, myoglobin is metabolized into bilirubin, which is converted to urobilinogen by gut bacteria [16,17]. Proteinuria can be attributed to the myoglobin release and degradation of other proteins from myocytes [16]. In the setting of elevated CK level in our patient, she was likely releasing large amounts of myoglobin that was degraded into bilirubin, which likely contributed to the presence of elevated urobilinogen in the urine. Despite the lack of urine hemoglobin and myoglobin, the presence of proteinuria and urobilinogen can serve as a proxy for determining myoglobinuria.

One study has suggested that pre-existing asthma is not a significant risk factor for contracting COVID-19 but having an acute exacerbation within the past year is associated with higher mortality [18]. Review of the patient’s chart reveals that she had an acute asthma exacerbation just one month prior to contracting COVID-19. Studies have suggested that ACE2 expression is reduced in asthmatics due to the Th2-mediated inflammation of epithelial cells, which could decrease susceptibility to COVID-19 [18]. However, poor asthma control and exacerbation place the patient in a proinflammatory state that could lead to a cytokine storm, thus increasing the risk of mortality from COVID-19 [18].

There is one clear predisposing factor for our patient, which is morbid obesity. The state of chronic inflammation seen in obese patients predisposes them to cytokine storm seen in severe COVID-19 infection [19]. Furthermore, obese patients are at a higher risk of contracting COVID-19 due to the higher expression of the ACE2 enzyme in adipose tissue [19]. Intriguingly, our patient is both morbidly obese and asthmatic, so it is difficult to determine whether her ACE2 expression is higher or lower than average. Regardless, her morbid obesity and recent asthma exacerbation likely had synergistic effects that increased the disease severity and poor outcome.

One limitation of this case report is the lack of urine myoglobin level in our patient. As discussed above, the presence of elevated urinary protein and urobilinogen can be used to determine myoglobinuria in the setting of myalgias and elevated CK. Alternatively, we could directly check urine myoglobin levels in patients who present with myalgias, but this may be impractical due to additional financial burden. Another limitation is the lack of a formal workup for a possible underlying autoimmune disease. Given that our patient is a young African American female, she fits the demographic that has a higher probability of having an undiagnosed autoimmune disorder which could have contributed to her robust inflammatory response to COVID-19. Since rhabdomyolysis can have a vague initial presentation, it may be prudent to check serum CK levels in patients that test positive for COVID-19, particularly in obese patients. If serum CK level exceeds >1000 U/L and rhabdomyolysis is diagnosed, then conservative treatment with intravenous fluids can prevent the development of acute kidney injury and help reduce the disease burden. Current literature suggests that elevated CK with or without overt rhabdomyolysis is associated with increased mortality among COVID-19 patients, but the limited sample size leaves room for more investigation [9,10]. As a scientific community, it is our responsibility to determine which patients are at a higher risk of developing severe COVID-19 complications. It may be reasonable to include the serum CK level in the initial workup of COVID-19, and if this level is high, then it could alert for more aggressive measures to help prevent future comorbidities.

## Conclusions

Rhabdomyolysis is a rare finding associated with COVID-19 that can have an early or late presentation. It is predominantly seen in middle-aged obese males, but it can be seen in younger females as well. The presence of elevated CK has been associated with higher mortality among COVID-19 patients, but current literature demonstrates that the majority of these patients are older males. There is no causal relationship between rhabdomyolysis and poor prognosis for COVID-19. However, it is still imperative to recognize and treat rhabdomyolysis in all patients, particularly younger females, to help mitigate the comorbidities of COVID-19.
